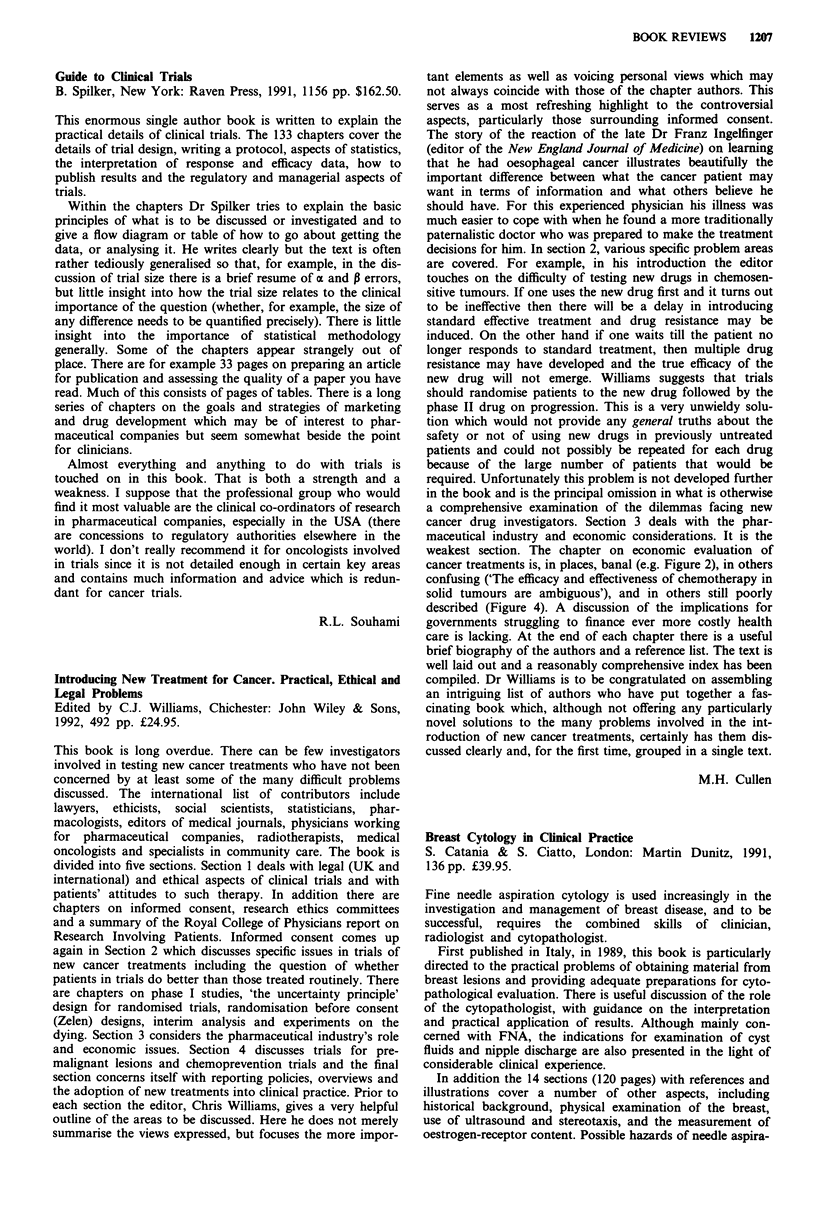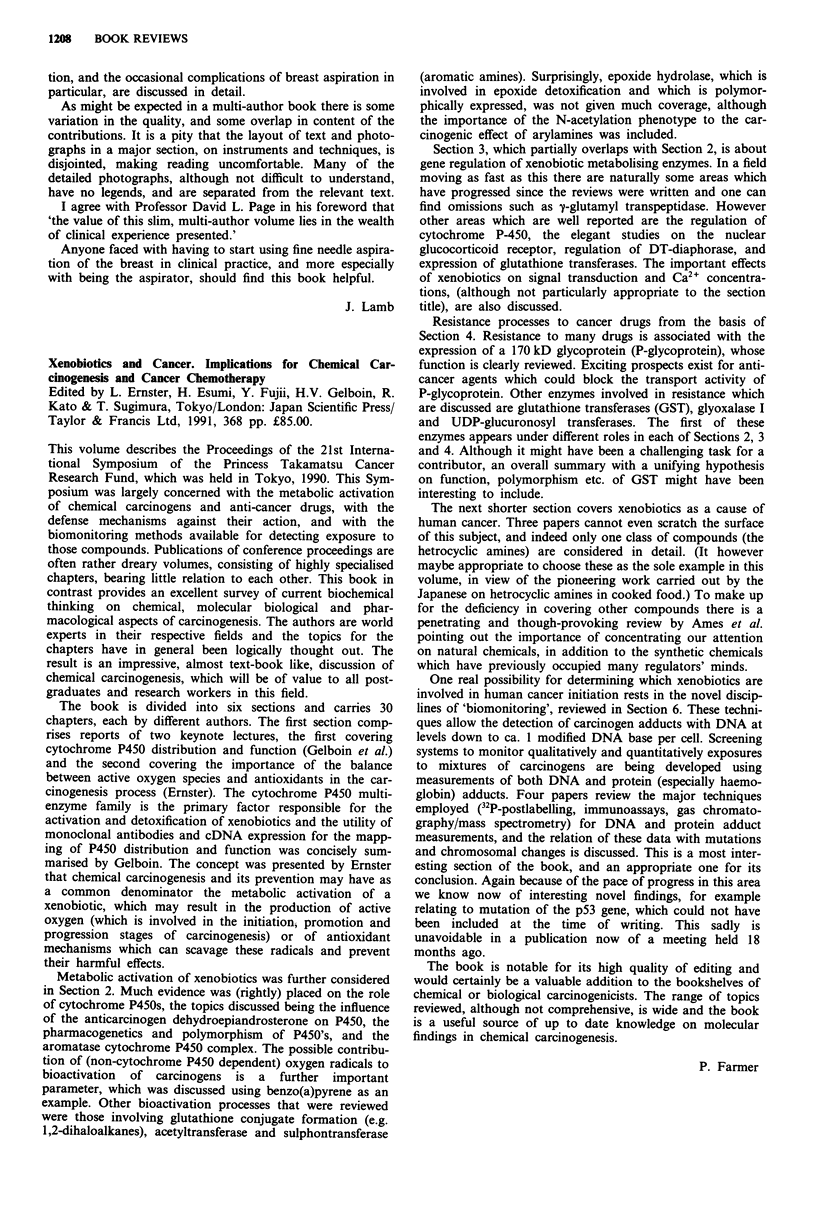# Breast Cytology in Clinical Practice

**Published:** 1992-12

**Authors:** J. Lamb


**Breast Cytology in Clinical Practice**
S. Catania & S. Ciatto, London: Martin Dunitz, 1991, 136 pp. £39.95.

Fine needle aspiration cytology is used increasingly in the investigation and management of breast disease, and to be successful, requires the combined skills of clinician, radiologist and cytopathologist.

First published in Italy, in 1989, this book is particularly directed to the practical problems of obtaining material from breast lesions and providing adequate preparations for cytopathological evaluation. There is useful discussion of the role of the cytopathologist, with guidance on the interpretation and practical application of results. Although mainly concerned with FNA, the indications for examination of cyst fluids and nipple discharge are also presented in the light of considerable clinical experience.

In addition the 14 sections (120 pages) with references and illustrations cover a number of other aspects, including historical background, physical examination of the breast, use of ultrasound and stereotaxis, and the measurement of oestrogen-receptor content. Possible hazards of needle aspiration, and the occasional complications of breast aspiration in particular, are discussed in detail.

As might be expected in a multi-author book there is some variation in the quality, and some overlap in content of the contributions. It is a pity that the layout of text and photographs in a major section, on instruments and techniques, is disjointed, making reading uncomfortable. Many of the detailed photographs, although not difficult to understand, have no legends, and are separated from the relevant text.

I agree with Professor David L. Page in his foreword that 'the value of this slim, multi-author volume lies in the wealth of clinical experience presented.'

Anyone faced with having to start using fine needle aspiration of the breast in clinical practice, and more especially with being the aspirator, should find this book helpful.

J. Lamb